# Supplementary material for: Hot spot prediction in protein-protein interactions by an ensemble system
Source: BMC Syst Biol. 2018 Dec 31;12(Suppl 9):132. doi: 10.1186/s12918-018-0665-8 (PMC6311905; doi:10.1186/s12918-018-0665-8)
Supplement: Supplementary file 2 — All relevant MATLAB code. A simple Matlab implement of our predictor. (ZIP 19,742 kb) [file 12918_2018_665_MOESM2_ESM.zip › Additional file 2/Read ASA/ASA Property/SKEMPI/4CPAI.txt]

 NetSurfP - Protein Surface Accessibility and Secondary Structure Predictions

  

|  |  |  |
| --- | --- | --- |
|  |  | NetSurfP - Protein Surface Accessibility and Secondary Structure Predictions  Technical University of Denmark |

  


---

```
# For publication of results, please cite:
# A generic method for assignment of reliability scores applied to solvent accessibility predictions.
# Bent Petersen, Thomas Nordahl Petersen, Pernille Andersen, Morten Nielsen and Claus Lundegaard
# BMC Structural Biology 2009, 9:51 doi:10.1186/1472-6807-9-51
#
# Column 1: Class assignment - B for buried or E for Exposed - Threshold: 25% exposure, but not based on RSA
# Column 2: Amino acid
# Column 3: Sequence name
# Column 4: Amino acid number
# Column 5: Relative Surface Accessibility - RSA
# Column 6: Absolute Surface Accessibility
# Column 7: Z-fit score for RSA prediction
# Column 8: Probability for Alpha-Helix
# Column 9: Probability for Beta-strand
# Column 10: Probability for Coil
E Q  4CPAI                  1    0.886 158.186   0.782   0.003   0.003   0.994
E H  4CPAI                  2    0.619 112.614  -1.468   0.058   0.017   0.925
E A  4CPAI                  3    0.480  52.929  -1.552   0.113   0.043   0.844
E D  4CPAI                  4    0.540  77.828  -0.630   0.115   0.016   0.868
E P  4CPAI                  5    0.438  62.095  -0.518   0.184   0.043   0.773
B I  4CPAI                  6    0.244  45.196  -0.685   0.184   0.043   0.773
B C  4CPAI                  7    0.158  22.155  -0.486   0.113   0.043   0.844
E N  4CPAI                  8    0.636  93.169  -0.411   0.113   0.043   0.844
E K  4CPAI                  9    0.540 111.016   0.666   0.058   0.017   0.925
E P  4CPAI                 10    0.444  62.961  -0.239   0.018   0.019   0.964
B C  4CPAI                 11    0.100  14.054  -0.984   0.018   0.019   0.964
E K  4CPAI                 12    0.552 113.526   0.344   0.052   0.084   0.864
E T  4CPAI                 13    0.433  60.002  -0.038   0.052   0.084   0.864
E H  4CPAI                 14    0.381  69.231  -0.728   0.053   0.043   0.903
E D  4CPAI                 15    0.679  97.815  -1.660   0.053   0.043   0.903
E D  4CPAI                 16    0.468  67.496  -0.558   0.053   0.043   0.903
B C  4CPAI                 17    0.050   7.034  -0.375   0.113   0.043   0.844
E S  4CPAI                 18    0.371  43.446   0.152   0.184   0.043   0.773
B G  4CPAI                 19    0.122   9.617  -1.286   0.386   0.097   0.517
B A  4CPAI                 20    0.110  12.067  -0.212   0.538   0.173   0.289
B W  4CPAI                 21    0.094  22.583  -0.037   0.662   0.225   0.113
B F  4CPAI                 22    0.060  12.022   0.362   0.662   0.225   0.113
B C  4CPAI                 23    0.019   2.738   0.847   0.807   0.137   0.056
B Q  4CPAI                 24    0.218  39.006   0.551   0.831   0.044   0.125
B A  4CPAI                 25    0.121  13.334  -0.388   0.831   0.044   0.125
B C  4CPAI                 26    0.032   4.507  -0.468   0.751   0.050   0.199
B W  4CPAI                 27    0.091  21.910  -0.396   0.717   0.014   0.269
E N  4CPAI                 28    0.457  66.919  -0.548   0.522   0.016   0.462
E S  4CPAI                 29    0.367  43.036  -0.577   0.430   0.016   0.555
B A  4CPAI                 30    0.189  20.850  -2.357   0.339   0.016   0.645
E R  4CPAI                 31    0.431  98.630  -0.158   0.339   0.016   0.645
E T  4CPAI                 32    0.466  64.690  -0.317   0.257   0.016   0.727
B C  4CPAI                 33    0.180  25.328  -1.784   0.184   0.043   0.773
E G  4CPAI                 34    0.375  29.497  -1.588   0.058   0.017   0.925
E P  4CPAI                 35    0.517  73.348  -1.163   0.018   0.019   0.964
E Y  4CPAI                 36    0.478 102.170  -0.877   0.018   0.047   0.935
E V  4CPAI                 37    0.685 105.223  -1.492   0.003   0.003   0.994

Explain the output.  Go back.
```

---
